# Development and validation of a screening tool for early identification of bloodstream infection in older patients – a retrospective case-control study

**DOI:** 10.1186/s12877-019-1402-x

**Published:** 2020-01-03

**Authors:** Sandra A. N. Walker, Heather Bannerman, Nathan Ma, Christine Peragine, Marion Elligsen, Lesley Palmay, Evelyn Williams, Barbara Liu

**Affiliations:** 1 Department of Pharmacy, Sunnybrook Health Sciences Centre, 2075 Bayview Avenue, Toronto, ON M4N 3M5 Canada; 2 Leslie L. Dan Faculty of Pharmacy, University of Toronto, Toronto, Canada; 3 Division Long-Term Care, Sunnybrook Health Sciences Centre, Toronto, Canada; 4 Faculty of Medicine, University of Toronto, Toronto, Canada

**Keywords:** Older, Elderly, Bacteremia, Predictive tool, Screening tool, Bloodstream infection(s)

## Abstract

**Background:**

Delayed diagnosis of bloodstream infection (BSI) occurs in > 20% of older patients, with misdiagnosis in 35%. Our objective was to develop and validate a clinically useful screening tool to identify older patients with a high probability of having a BSI.

**Methods:**

Hospitalized patients > 80 years old with BSI (*n* = 105/group) were evaluated for the tool development in this retrospective matched case-controlled study (learn cohort). The tool was validated in different retrospectively matched case and control patients > 80 years old (*n* = 120/group) and 65 to 79 years old (*n* = 250/group) (test cohort). Binary logistic regression was used to develop a screening tool using laboratory and clinical parameters that were significantly associated with BSI (*P* < 0.05; adjusted odds ratio (OR) > 1); and Classification and Regression Tree (CART) analysis was used to identify parameter breakpoints. Performance metrics were used to evaluate and validate the tool.

**Results:**

The significant parameters associated with BSI were maximum temperature (Tmax)(> 37.55C)(OR = 42.575), neutrophils (> 7.95)(OR = 1.923), a change in level of consciousness (LOC) (Yes = 1, No = 0)(OR = 1.571), blood urea nitrogen (BUN)(> 10.05)(OR = 1.359), glucose (> 7.35)(OR = 1.167), albumin (< 33.5)(OR = 1.038) and alanine aminotransferase (ALT) (> 19.5)(OR = 1.005). The optimal screening tool [Ln (odds of BSI) = − 150.299 + 3.751(Tmax) + 0.654(neutrophils) + 0.452(change in LOC) + 0.307(BUN) + 0.154(glucose) + 0.038(albumin) + 0.005(ALT)] had favorable performance metrics in the learn and test cohorts (sensitivity, specificity and accuracy of 95% in the learn cohort and 77, 89, and 81% in the total test cohort); and performed better than using only temperature and neutrophil count.

**Conclusions:**

The validated tool had high predictive value which may improve early identification and management of BSI in older patients.

## Background

While older patients are at increased risk of infection, typical manifestations of infection in the elderly are more subtle, or nonexistent [1–3]. Although fever is a principal sign of infection in other patients populations, it may be absent or diminished in 20–30% of older patients with severe infections [1, 2]. Diagnosing infection in the elderly may be complicated by a non-specific clinical presentation (e.g. falls, altered mental status, delirium, anorexia, loss of urinary or fecal continence, and weakness [1, 3–6]) which may mimic non-infectious syndromes [1, 2]. Consequently, a delay in diagnosis of bacteremia in geriatric patients occurs in more than 20% of cases, with misdiagnosis in 35% [7]. Therefore, improvement in the early identification and treatment of bacteremia in aging patients is needed.

Bloodstream infections (BSI) are associated with significant morbidity and mortality in the elderly population [8]; with case-fatality rates ranging from 40 to 60% [7]. Therefore, healthcare practitioners must improve their clinical suspicion for BSI in their elderly patients so that they can appropriately draw blood cultures and begin early empiric antibiotic treatment [4].

Our objective was to develop and validate a clinically useful screening tool to identify older patients with a high probability of having a BSI to improve early diagnosis and management of these patients.

## Methods

### Location

This study was conducted at Sunnybrook Health Sciences Centre (SHSC), a 1359 bed tertiary care teaching hospital in Toronto, Ontario, Canada. The study was approved with a waiver for the need of informed consent by the SHSC Research Ethics Board on February 3, 2014.

### Study design

**Fig. 1:**
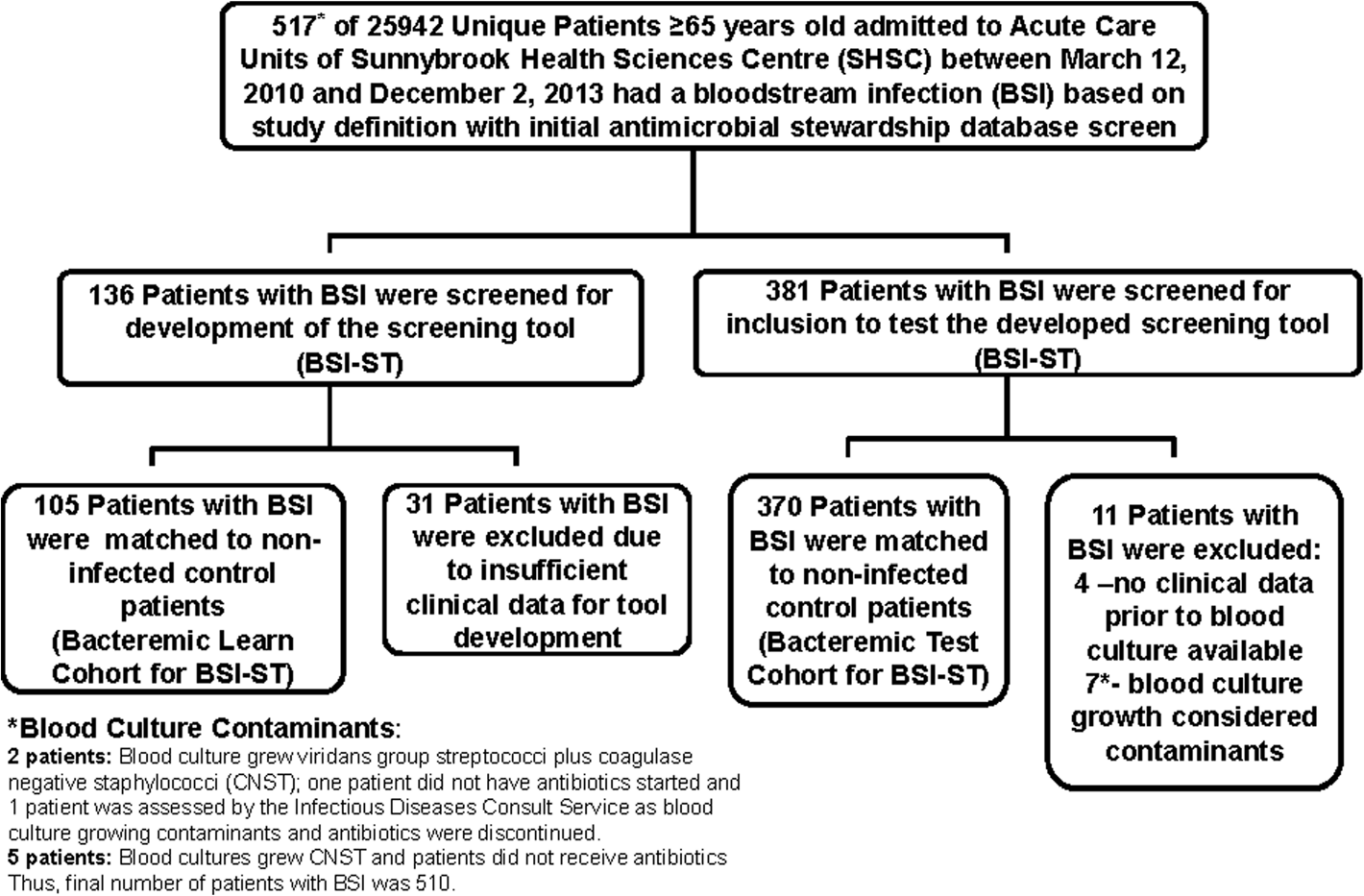
Patient Inclusion. Case patients were those with a positive blood culture, excluding those with coagulase-negative *Staphylococci*, *Corynebacterium*, *Propionibacterium*, and *Bacillus* species other than *B. anthracis*, in whom antimicrobials were begun. Non-infected Matched Controls were older patients who never had a positive culture (any site) and did not receive any antibiotics during their hospital stay who were matched to cases by sex, age (within 5 years), hospital location (critical care [level II or III] or ward), length of stay and date of stay at the matching hospital location (within 30 days) at Sunnybrook Health Sciences Centre

This was a retrospective case-control study of hospitalized older patients (≥80 years of age on admission for the tool development (learn) cohort and the first validation (test) cohort; and > 65–79 years of age for the second validation (test) cohort) admitted to SHSC between March 12, 2010 and December 2, 2013. Patients were identified for study inclusion using the electronic Sunnybrook Antimicrobial Stewardship Program (ASP) database which automatically accesses and stores all microbiology, laboratory, pharmacy, and admissions, discharges and transfer data for all hospitalized patients at SHSC. Control patients were matched to case patients with identified BSI based on sex, age (within 5 years), hospital location (critical care [level II or III] vs ward), length of stay, and date of stay (within 30 days) at SHSC. Relevant data were extracted from the patients’ health records by a single reviewer for the learn cohort (HB) and test cohorts (> 80 years old (CP), 65–79 year old (NM)). A total of 105 matched pairs (*n* = 210) of patients were included in this study to develop the BSI screening tool (learn cohort) (Fig. 1). These pairs were randomly chosen (using a random number generator) from the eligible patient population identified by the ASP database. All remaining eligible older patients admitted during the study period (370 matched cases (*n* = 740)) were used as the test cohorts to validate the screening tool (120 retrospective matched cases > 80 years old (*n* = 240) and 250 retrospective matched cases (*n* = 500) between the ages of 65–79 years). The evaluation of the tool in the younger test cohort (65–79 years) was completed to determine the generalizability (robustness) of the developed BSI tool.

### Data collection

Cohort definitions and patient parameters extracted for the learn and test cohorts for evaluation are provided in the Additional file 1.

### Statistics

A sample size of 105 patients in each of the case and control learn groups allowed for assessment of 10 (at a ratio of 10:1) up to 50 (at a ratio of 2:1) independent variables for association with presence of BSI (dependent variable) and are within acceptable ratio limits [9–14].

Laboratory or clinical parameters which had data available for < 20% of study patients were excluded from all statistical analyses. For comparisons of cases versus controls, a Fisher’s exact test, Mann-Whitney test, or two-tailed unpaired t-test, with or without Welch correction, was used for nominal, ordinal or nonparametric interval data, and parametric interval data with unequal or equal standard deviations, respectively. Interval data were assessed for normality using the Kolmogorov-Smirnov test. A *P*-value of < 0.05 was considered statistically significant. Nominal data were reported as number (%), ordinal and nonparametric interval data were reported as median (range), and parametric interval data were reported as mean (± standard deviation (SD)). Univariate analysis and Pearson’s Correlation Matrix (SPSS®) were conducted in the learn patient cohort to identify laboratory or clinical parameters which were significantly (*P* < 0.05) associated with BSI.

Statistically significant (*P* < 0.05) parameters following univariate analysis, with confirmation by Pearson’s Correlation Matrix, for which data were available for ≥20% of study patients in the learn cohort were then analysed using binary logistic regression (BLR) (SPSS®). An iterative process was used to identify the simplest BLR model with the highest overall predictability that was statistically significant (*P* < 0.05) in which all independent variables predicting BSI input into the regression model were statistically significant on the iterative model entry (*P* < 0.05) and had an adjusted odds ratio > 1 in the resulting model. A Receiver Operating Characteristic (ROC) curve was developed to identify the decision threshold value for the probability of BSI for the BLR equation that would optimize the regression equation’s predictive value (i.e. maximize sensitivity while minimizing the false positive rate of the binary logistic regression equation) and the area under the ROC was determined using the trapezoidal rule. The probability of BSI for each case and control as determined from the identified best regression model was calculated from the regression equation as follows: the regression equation provided the Ln (odds of BSI), therefore the probability of BSI = Odds of BSI / (1+ Odds of BSI). Classification and regression tree (CART) analysis (Salford Predictive Modeling Suite®) was used to identify breakpoints of each independent variable that remained significant in the BLR model. Sensitivity and specificity analyses were conducted on the BLR equation with optimal probability of infection threshold, the CART model, and the CART breakpoints to determine the optimal screening tool. Metrics calculated in the sensitivity and specificity analyses were sensitivity, specificity, accuracy, positive predictive value (PPV), negative predictive value (NPV), positive post-test probability, negative post-test probability (NPTP), false positive rate (FPR), false negative rate (FNR), positive likelihood ratio (LR+), and negative likelihood ratio (LR-). The optimal screening tool developed in the learn elderly population was defined as the one with the overall best combination of highest sensitivity, specificity, accuracy, positive and negative predictive value, and positive likelihood ratio; and lowest negative likelihood ratio, negative post- test probability, false positive rate and false negative rate. The optimal screening tool was then validated with the same sensitivity and specificity metrics in a retrospective cohort of 370 different eligible matched cases (*n* = 740) analysed for patients ≥65 years old with sub-analyses for > 80 years old and between 65 and 79 years old. All metric calculations completed for the learn and test cohorts were based on the corresponding data from those cohorts.

## Results

A total of 25,942 unique patients ≥65 years old were admitted to acute care units at SHSC, between March 12, 2010 and December 2, 2013 and 510 had BSI. (Fig. 1) Thus, the period prevalence of BSI in patients > 65 years of age at SHSC was 2.0% (510/25942). Of the 256 patients > 80 years old with BSI, 105 patients and their matched controls were randomly chosen for the development of the BSI screening tool (learn cohort). The patient characteristics of the learn cohort are summarized in Table 1. Types of bacteria and sources of BSI for the learn cohort are summarized in Additional file 1: Table S1.

**Table 1:** Learn Cohort Patient Characteristics

Parameter	Total (%)	Case (%)	Control (%)	*p*-value	OR	95% CI for OR
Number of patients	210	105 (50)	105 (50)	–	–	–
Sex (Male)	108 (51)	54 (51)	54 (51)	1.11	1.00	0.58–1.72
Mean Age on Date of Study Entry (Years) (± SD)	86 (± 4)	86 (± 4)	85 (± 4)	0.70	–	–
Hospital Location (Ward)	208 (99)	104 (99)	104 (99)	1.50	1.00	0.06–16.21
Median LOS (Days) at Study Entry (Range)	0.31 (0–27)	0.31 (0–27)	0.31 (0–27)	> 0.999	–	–
Neoplastic Disease	82 (39)	42 (40)	40 (38)	0.89	1.08	0.62–1.89
Diabetes Mellitus	56 (27)	29 (28)	27 (26)	0.88	1.10	0.60–2.03
Dementia	55 (26)	28 (27)	27 (26)	1.00	1.05	0.57–1.94
CHF	60 (29)	29 (28)	31 (30)	0.88	0.91	0.50–1.66
COPD	29 (14)	14 (13)	15 (14)	1.00	0.92	0.42–2.02
ESRD	11 (5)	8 (8)	3 (3)	0.21	2.80	0.72–10.89
Malnutrition	6 (3)	2 (2)	4 (4)	0.66	0.45	0.07–2.76
Antipyretic Use^a^	62 (30)^b^	37 (36)^b^	25(24)	0.07	1.79	0.98–3.28
Corticosteroid Use^c^	28 (13)	10 (10)	18 (17)	0.15	0.51	0.22–1.16
Survival	194 (92)	91 (87)	103 (98)	0.003	0.13	0.03–0.57

^a^Defined as any antipyretic medication given on the day of patient data study entry date

^b^Medication administration record to identify antipyretic use on day of patient study entry date was not found by Health Data Resources for two Case patients. Therefore, denominator used for Cases and Total in this analysis were 103 and 208, respectively

^c^Defined as any corticosteroid medication given within 7 days of patient data study entry date

Following univariate analysis, parameters that were significantly (*P* < 0.05) different between case patients and their matched controls in the learn cohort were: white blood cell count (WBC), Polymorphonuclear neutrophil (PMN), blood urea nitrogen (BUN), serum creatinine, aspartate aminotransferase (AST), alanine aminotransferase (ALT), glucose, lowest heart rate, highest heart rate, minimum temperature (Tmin), maximum temperature (Tmax), chills, vomiting, change in level of consciousness (∆ LOC), change in mental status, fecal incontinence, albumin, lowest systolic blood pressure (SBP), lowest diastolic blood pressure (DBP), presence of central venous catheter within 24 h of study day, gastrostomy within 24 h of study day, and survival. Laboratory or clinical parameters which were available for < 20% of study patients and which were excluded from the analysis were: C-reactive protein (CRP), Erythrocyte sedimentation rate (ESR), ferritin, lactate, and change in mental status. Therefore, a total of 20 significant independent variables with ≥20% patient data (i.e. all significant variables via univariate analysis except survival and change in mental status) were used in the initial BLR model inputs (patient:variable ratio of 5:1).

**Fig. 2:**
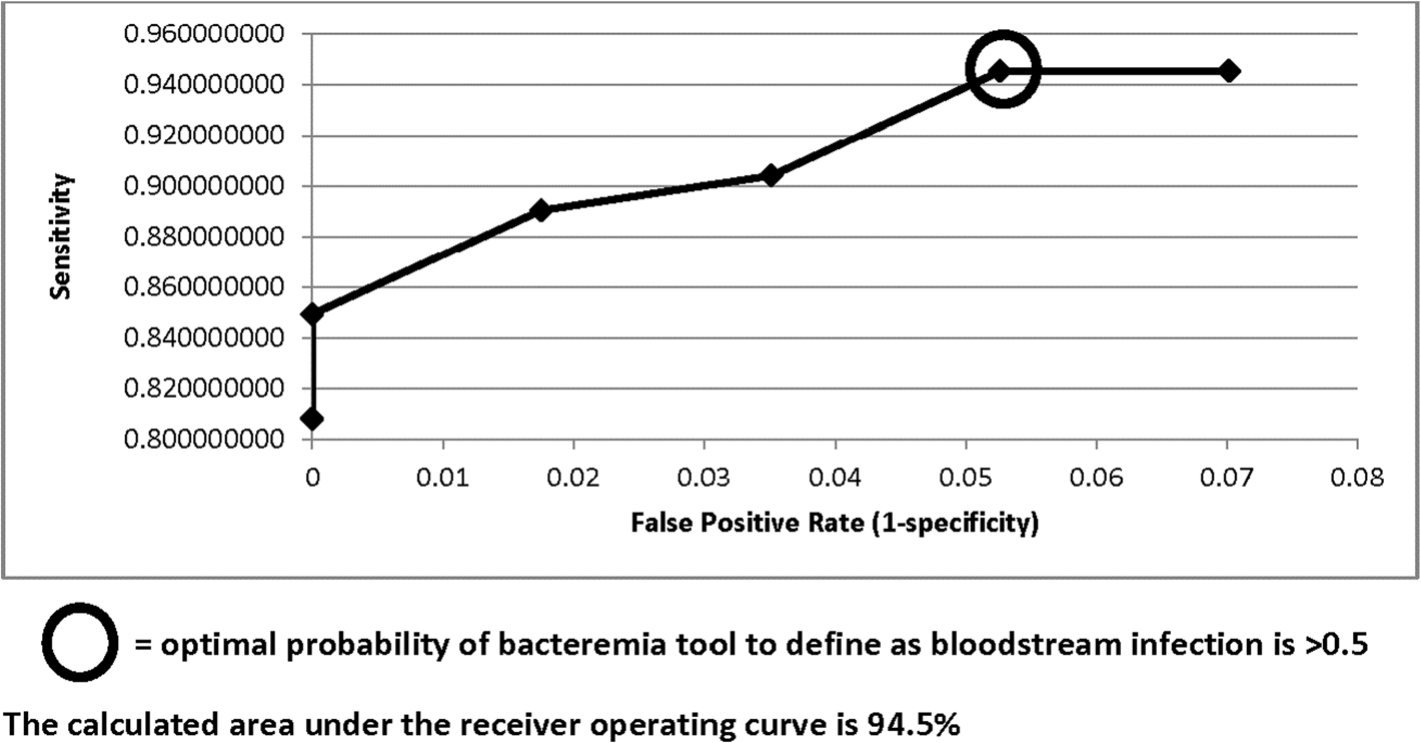
Receiver Operating Curve Identifying Optimal Binary Logistic Regression Bloodstream Infection Tool Breakpoint for Bloodstream Infection

After an iterative process using BLR, the best model identified was statistically significant (*P* < 0.0001). Each independent variable included in this model was statistically significant in the previous model iteration at model entry (*P* < 0.05), and had an OR > 1 in the final model logistic regression equation. Patients with missing model variables are removed by the SPSS BLR analysis so that the resultant number of patients included in the best regression model was 130 (73 with infection and 57 controls). The optimal BLR model equation was: ***Ln (odds of BSI) = − 150.299 + 3.751(Tmax;*** ^***o***^***C) + 0.654(PMN; × 10***^***9***^***/L) + 0.452(∆ LOC; Yes = 1,No = 0***) ***+ 0.307(BUN; mmol/L) + 0.154(glucose; mmol/L) + 0.038(albumin; g/L) + 0.005(ALT; U/L)***. The adjusted odds ratios, confidence intervals, and CART determined breakpoints for these variables are summarized in Table 2. The receiver operating characteristic (ROC) curve identified decision threshold value which optimized the predictive value of the regression model to designate a patient as having a BSI was a probability of BSI cut-off of > 0.5 (Fig. 2). The area under the ROC curve was calculated to be 94.5%.

**Table 2:** Variables in Final Binary Logistic Regression Equation Bacteremia Screening Tool^a^

Independent Variable	OR	95% CI	CART Breakpoint for Association with Bacteremia^b^
Maximum Temperature (Tmax, ^o^C)	42.575	5.358–338.322	> 37.55
Neutrophils (PMN)	1.923	1.290–2.868	> 7.95
Change in Level of Consciousness (LOC) (Yes)	1.571	0.238–10.370	1
Blood Urea Nitrogen (BUN)	1.359	1.115–1.657	> 10.05
Glucose	1.167	0.714–1.906	> 7.35
Albumin	1.038	0.873–1.235	≤33.5
Alanine Aminotransferase (ALT)	1.005	0.992–1.019	> 19.5

^a^*Ln (odds of BSI) = −150.299 + 3.751(Tmax;* ^*o*^*C) + 0.654(PMN; ×10*^*9*^*/L) + 0.452(∆ LOC; Yes = 1,No = 0) + 0.307(BUN; mmol/L) + 0.154(glucose; mmol/L) + 0.038(albumin; g/L) + 0.005(ALT; U/L)*

^b^When all variables input and parent node is Tmax

The optimal model predicting BSI was the BLR equation with a threshold probability of infection cut-off of > 0.5 (Additional file 1: Table S2). Sensitivity and specificity analysis of the optimal BLR derived model, with a threshold probability cut-off defining BSI of > 0.5 (or > 50%) in the learn population had a sensitivity, specificity, and accuracy of 95% each (Additional file 1: Table S2). The risk of missing a BSI (that is the false negative rate) was only 5.5% and the false positive rate was only 5.3%.

Validation of this model in the test cohort of elderly patients ≥65 years old (486 patients with a complete data set, BSI cohort = 294 and Control cohort = 192) identified that this tool has a sensitivity, specificity and accuracy of 77, 89 and 81%, respectively (Additional file 1: Table S3). In order to generalize the findings of this study to institutions with a different pre-test probability (prevalence) of BSI in their elderly patients (≥65 years of age), Additional file 1: Table S4 is provided to identify a range of pre-test probabilities from 1 to 100% with the corresponding PPV, NPV and NPTP based on the metrics from the validated cohort of patients ≥65 years old. At the institutional period prevalence of BSI in patients > 65 years of age of 2.0%, this would mean that the risk of missing a BSI in a patient that tested negative with the BSI screening tool would only be 0.5% (Additional file 1: Table S4).

Although temperature and neutrophil count were the parameters of highest weighting in the BSI tool; if only these parameters were used to predict bacteremia in the learn cohort (Tmax > 37.55 and PMN > 7.95), the sensitivity and accuracy would drop to 52 and 75%, respectively, and the false negative rate would rise to 48%. The specificity and false positive rate would be 99 and 1%, respectively. If only temperature and neutrophil count (Tmax > 37.55 and PMN > 7.95) were used in the test cohort of patients > 65 years of age, the sensitivity and accuracy would drop to 37 and 68%, respectively, and the FNR would rise to 63%. The specificity and FPR would be 99 and 1%, respectively.

## Discussion

**Fig. 3:**
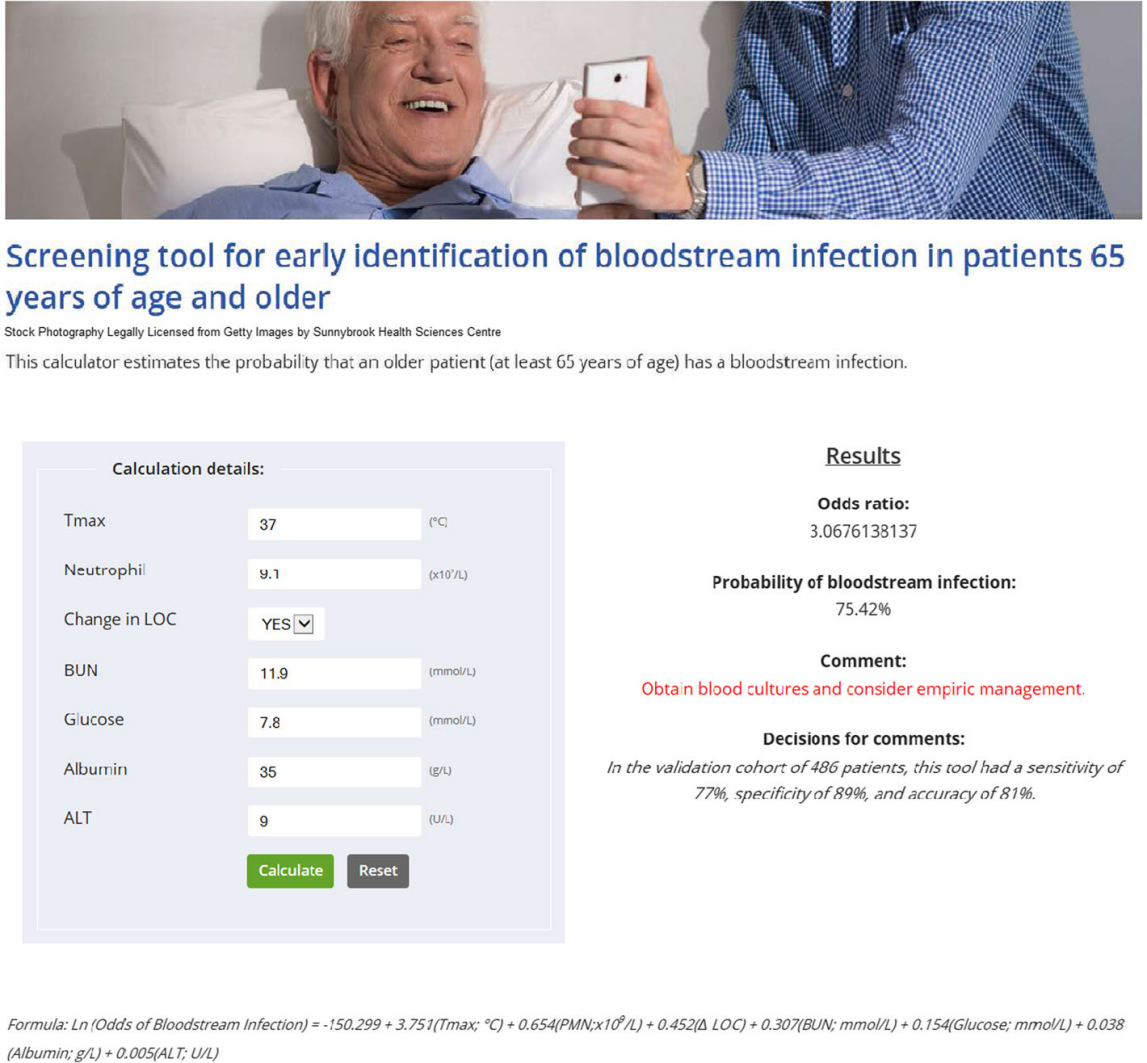
App for Determining Probability of Bloodstream Infection in Older Patients

The objective of this study was to develop and validate a clinically useful screening tool to identify older patients with a high probability of having a BSI to improve early diagnosis and management of these patients. The optimal screening tool for BSI in the elderly was derived by BLR; whereby the ***Ln (odds of BSI) = − 150.299 + 3.751(Tmax;*** ^***o***^***C) + 0.654(PMN; × 10***^***9***^***/L) + 0.452(∆ LOC; Yes = 1,No = 0***) ***+ 0.307(BUN; mmol/L) + 0.154(glucose; mmol/L) + 0.038(albumin; g/L) + 0.005(ALT; U/L)***. The ROC area under the curve was excellent for a screening tool at 94.5% and the ROC derived optimal breakpoint to conclude BSI with the tool was a probability of BSI of > 0.5. This tool had a sensitivity, specificity and accuracy of 95% in the learn population and 77, 89, and 81% in the test population. The risk of missing an elderly patient with a BSI was only 5.5% in the learn population. Although the FNR was 23% in the test population; at the institutional period prevalence of BSI in older patients of 2.0%, the probability of missing a BSI when the test result is negative (NPTP) is only 0.5%. A user-friendly app (available free of charge at https://sunnybrook.ca/content/?page=55562&pre=view) for clinicians to use this screening tool in their practice was developed to provide clinicians with a fast calculation of the probability of BSI (%) in their patients and make recommendations for obtaining blood cultures and consideration of empiric antimicrobial management based on practical probability cut-offs (Fig. 3). Although temperature and neutrophil count were the parameters of highest weighting in the BSI tool; if only these parameters were used to predict bacteremia in the learn cohort (Tmax > 37.55 and PMN > 7.95), there would be a high risk of missing an elderly patient with bacteremia with an intolerable false negative rate of 63% seen in the test cohort of this study.

This study has several strengths. The BSI screening tool was developed and validated in a large cohort of older patients. The large cohort of learn patients allowed an assessment of up to 50 potential independent variables for association with BSI [9–14] (patient:variable ratio 2:1), and a total of only 20 independent variables were input in the initial BLR analyses corresponding to a higher patient to variable ratio of 5:1. To minimize potential confounding variables, the cases of identified BSI were matched to controls based on sex, age (within 5 years), hospital location (critical care vs ward), length of stay, and date of stay (within 30 days) at SHSC. The final optimal BSI screening tool is very convenient to use with a freely accessible app that provides clinicians with the calculation of the probability of BSI and guidelines for next steps in patient care. The clinical and laboratory parameters in the optimal BSI tool are readily available and routinely ordered during a patient’s hospital stay (Tmax, PMNs, BUN, glucose, albumin, and ALT). Of importance, none of the laboratory parameters in the final BSI screening tool require the clinician to have a pre-existing suspicion of infection (e.g. lactate, CRP, ESR); since the whole point and value of the BSI screening tool is to enable early identification of older patients with a high probability of BSI in whom blood cultures and other confirmatory laboratory parameters (e.g. lactate, CRP, ESR) should be drawn. The optimal BSI screening tool has excellent predictive capability as demonstrated in both learn and test cohorts of patients. We developed a table (Additional file 1: Table S4) to enable generalizability of the tool to hospitals that may have a different prevalence of BSI in their older patients ≥65 years of age in order to provide them with a calculated probability of missing a BSI in patients with a negative test result (i.e. NPTP) along with the negative and positive predictive values. Due to the severe consequences of a false negative result in this patient population, a predictive tool with a low NPTP is desirable. Even at a high pre-test probability (prevalence) of bacteremia of 30%, the NPTP of our BSI screening tool is calculated to be only 10%. In a population with a lower prevalence of bacteremia, such as the population studied by Fontanarosa et al. [7] who found a prevalence of bacteremia of about 10%, our validated tool would have a NPTP of 3%, compared to the un-validated tool by Fontanarosa which had a NPTP of 4% in the learn population. If, like Fontanarosa et al. [7] we used the learn population to determine the metrics in our study, the calculated NPTP with a 10% prevalence of bacteremia is only 0.64% (data not shown). The only other study we found which developed a predictive tool for bacteremia in older patients [15] (un-validated tool) had a NPTP of 44% at a study BSI prevalence of 50%, which is unacceptably high. At the high pre-test probability (prevalence) of BSI of 60% in our test cohort, our PPV is 91%. In Fontanarosa’s [7] patient population with a bacteremia prevalence of about 10%, our PPV in the learn and test population would be 67% (data not shown) and 44%, respectively; whereas the tool developed by Fontanarosa et al. [7] had a PPV of only 16%. To apply our tool to a real population, a study by Windsor [16] reported a 14% prevalence of bacteremia in a geriatric unit. If we used our validated tool results to predict a diagnosis of bacteremia in that population, our risk of missing a true bacteremia with a negative tool result would be only 4%. The tool that was developed and validated in our study demonstrated better predictive metrics, including a lower false negative and false positive rate compared to previously published and un-validated bacteremia screening tools in the elderly patient population [ 7, 8, 15, 17].

This study is not without limitations. The retrospective study design made some parameters (such as mental status change) difficult to assess. Additionally, some parameters were measured in < 20% of patients (CRP, lactate, ESR, and ferritin) and therefore had to be excluded from the multivariate analysis. However, absence of these parameters in our BSI screening tool strengthens the practical value of our tool for use in patients where the clinician has not yet ordered laboratory parameters associated with infection (e.g lactate, CRP, ESR), because the index of suspicion for infection does not yet exist and therefore, these laboratory parameters would not be available for the patient. Therefore, the tool allows clinicians to use commonly available laboratory parameters in most patients to make an initial assessment with the BSI screening tool, which would then trigger the ordering of appropriate laboratory parameters specifically associated with BSI (i.e. blood cultures, lactate, ESR, CRP). Our screening tool for bacteremia was developed in patients who were 80 years of age and older, since these are the patients in which the diagnosis of BSI may be most difficult. However, the screening tool was then validated in older patients ≥65 years old with excellent results. The patients in both learn and test cohorts were almost entirely on the ward; therefore, the application of this tool to critically ill patients is not known. We hope to evaluate the practical feasibility and performance metrics of the BSI screening tool developed in this study in a multicentre prospective study that includes elderly intensive care, emergency department and ward patients.

## Conclusions

This retrospective study developed and validated a practical and effective tool for predicting the presence of BSI in patients > 65 years old and may improve early diagnosis and management of these patients.

## Supplementary information

**Additional file 1: Table S1.** Learn Cohort Types and Sources of Bacteremia. **Table S2.** Comparison of Bacteremia Screening Tools Developed in Learn Cohort^a^. **Table S3.** Patient Demographics, Microbiology Summary and Tool Performance of Patients with Full Data Set Included in the Bloodstream Infection Tool Analyses. **Table S4.** What would happen to Positive Predictive Value, Negative Predictive Value and Negative Post-Test Probability if the true Pre-test Probability for Bacteremia in Older Patients was Higher or Lower than the Study Pre-Test Probability?^a^.

## Data Availability

The datasets used and/or analysed during the current study are available from the corresponding author on reasonable request.

## Abbreviations

∆ LOC: Change in level of consciousness
ALT: Alanine aminotransferase
ASP: Antimicrobial Stewardship Program
AST: Aspartate aminotransferase
BLR: Binary Logistic Regression
BSI(s): Blood stream infection(s)
BUN: Blood urea nitrogen
CART: Classification and Regression Tree
CRP: C-reactive protein
ESR: Erythrocyte sedimentation rate
FNR: False negative rate
FPR: False positive rate
LOC: Level of consciousness
LR-: Negative likelihood ratio
LR+: Positive likelihood ratio
NPTP: Negative post-test probability
NPV: Negative predictive value
PMN: Polymorphonuclear neutrophil
PPV: Positive predictive value
ROC: Receiver Operating Characteristic
SD: Standard deviation
SHSC: Sunnybrook Health Sciences Centre
Tmax: Maximum temperature
Tmin: Minimum temperature
WBC: White blood cell count
