# Supplementary material for: Development and validation of a screening tool for early identification of bloodstream infection in older patients – a retrospective case-control study
Source: BMC Geriatr. 2020 Jan 3;20:6. doi: 10.1186/s12877-019-1402-x (PMC6942330; doi:10.1186/s12877-019-1402-x)
Supplement: Supplementary file 1 — Additional file 1: Table S1. Learn Cohort Types and Sources of Bacteremia. Table S2. Comparison of Bacteremia Screening Tools Developed in Learn Cohorta. Table S3. Patient Demographics, Microbiology Summary and Tool Performance of Patients with Full Data Set Included in the Bloodstream Infection Tool Analyses. Table S4. What would happen to Positive Predictive Value, Negative Predictive Value and Negative Post-Test Probability if the true Pre-test Probability for Bacteremia in Older Patients was Higher or Lower than the Study Pre-Test Probability?a. [file 12877_2019_1402_MOESM1_ESM.docx]

**Development and Validation of a Screening Tool for**

**Early Identification of Bloodstream Infection in**

**Older Patients**

**SUPPLEMENTARY MATERIAL**

Sandra A.N. Walker PharmD^a,b *^, Heather Bannerman MD^a^, Nathan Ma PharmD^a,b^, Christine Peragine PharmD^a,b^, Marion Elligsen BScPhm^a^, Lesley Palmay MSc^a^,

Evelyn Williams MD^c,d^, Barbara Liu MD^c,d^

***Corresponding and Senior author:**

Dr. Sandra A.N. Walker

Department of Pharmacy

Sunnybrook Health Sciences Centre

2075 Bayview Avenue

Toronto, ON

M4N 3M5

Email address: [sandra.walker@sunnybrook.ca](mailto:sandra.walker@sunnybrook.ca)

Tel: 416-480-4494 Fax: 416-480-5877

Affiliations:

a. Department of Pharmacy, Sunnybrook Health Sciences Centre, Toronto, Canada

b. Leslie L. Dan Faculty of Pharmacy, University of Toronto, Toronto, Canada

c. Division Long-Term Care, Sunnybrook Health Sciences Centre, Toronto, Canada

d. Faculty of Medicine, University of Toronto, Toronto, Canada

**Data Collection**

Case patients in learn and test cohorts were those with a positive blood culture in whom antimicrobials were begun. Patients with positive blood cultures were excluded if they had blood isolates considered to represent contaminants (*Coagulase-negative Staphylococci, Corynebacterium, Propionibacterium*, and *Bacillus species* other than *B. anthracis*^8^ or had chart documented assessment by the hospital’s Infectious Diseases Consult service that the blood culture was considered a contaminant). Control patients in learn and test cohorts were older patients who never received antibiotics during their entire hospital stay and either never had cultures ordered or never had any positive culture from any source during their entire hospital stay. Included cases were matched to controls by gender, age (within 5 years), hospital location (critical care [level II or III] or ward), length of stay and date of stay at the matching hospital location (within 30 days) at SHSC. If patients had multiple positive blood cultures during a given hospital stay, data were only collected in relation to their first positive blood culture

**Learn Cohort**

The following parameters were determined within a period of 24 hours before day of positive blood culture in patients with BSI and the equivalent length of stay (LOS) in the uninfected matched control patient (i.e. if case patient had positive blood culture 72h after admission, then relevant parameters for case and their matched control patient were obtained from 48h to 72h after admission): gender; age; weight (kg); lowest and highest systolic blood pressure, heart rate, and temperature. The following laboratory parameters were determined within a period of 96 hours before or after day of positive blood culture in case patients or the equivalent LOS in the respective matched control patients (the closest value to the study day was collected with priority given to data collected before the date of blood culture draw in case patients): total white blood cell count (WBC), polymorphonuclear leukocytes (PMN), platelets, glucose, C-reactive protein (CRP), erythrocyte sedimentation rate (ESR), lactate, blood urea nitrogen (BUN), serum creatinine (sCr), aspartate aminotransferase (AST), alanine transaminase (ALT), alkaline phosphatase (ALP), bilirubin, albumin, prothrombin time (INR), partial thromboplastin time, thyroid stimulating hormone (TSH), ferritin, and results of all positive cultures (source and organism). The following clinical parameters were also collected within a period of 96 hours before or after day of first positive non-contaminant blood culture or the equivalent LOS in the respective matched control patients (unless otherwise stated): chills or rigors (Yes/No), vomiting (Yes/No), changing level of consciousness (defined as changes in the state of wakefulness of the patient determined by the strength of stimuli required to provoke responses^9^) (∆ LOC) (Yes/No), changes in mental status (defined as changes in behaviour, speech, motor activity, affect, mood, attitude, insight, thought, perception, and cognitive abilities^9^) (Yes/No). Additional clinical data collected were: presence of an indwelling urinary catheter in the 24 hour period prior to study date (Yes/No), central venous catheter present in the 24 hour period prior to study date (Yes/No), gastrostomy present in the 24 hour period prior to study date (Yes/No), surgery before culture in the current hospitalization (defined as any invasive medical procedure where there was a breech to the natural barrier of an organ system beyond routine medical care (ie. foley catheter, peripheral line, subcutaneous and intramuscular injections) (Yes/No), dysphagia (defined as the presence of a modified diet texture order in the patient chart unless clearly documented for another reason, ie. post-operative orders) (Yes/No), presence of a poor appetite (defined as documentation of poor appetite in the patient’s health record by a member of the interprofessional health care team), urinary incontinence (Yes/No), fecal incontinence (Yes/No), underlying neoplastic disease (Yes/No), diabetes mellitus (Yes/No), dementia (defined as a pre-existing diagnosis of irreversible cognitive impairment upon admission, including Alzheimer’s dementia, Vascular dementia, Lewy Body dementia, and frontotemporal dementia^10^) (Yes/No), congestive heart failure (Yes/No), chronic obstructive pulmonary disease (Yes/No), end stage renal disease (requiring dialysis) (Yes/No), severe malnutrition (defined as BMI <19 kg/m^2^ or severe wasting^11^) (Yes/No), corticosteroid use (intravenous, intramuscular, oral or inhaled Betamethasone, Beclomethasone, Budesonide, Ciclesonide, Cortisone, Hydrocortisone, Dexmethasone, Fludocortisone, Fluticasone, Methylprednisolone, Prednisolone, Prednisone, Triamcinolone acetonide) up to 7 days prior to study date) (Yes/No), antipyretic use on study date (Acetaminophen, ASA, Ibuprophen, Celecoxib, Diclofenac, Flurdiprofen, Ibuprofen, Indomethacin, Ketoprofen, Ketorolac, Mefenamic Acid, Meloxicam, Nepafenac, Naproxen, Prioxicam, Tiaprofenic Acid) (Yes/No) and survival (defined as hospital discharge or survival 14 days after study date) (Yes/No). The period of 96 hours before or after day of positive blood culture (patient with BSI) or the equivalent LOS in the respective matched control patients was chosen to avoid missing potential important variables that were infrequently ordered. Priority was given to parameter data that were available before blood culture draw for case patients and closest to the the equivalent LOS in the respective matched control patients. Potential cases and controls were excluded if WBC, PMNs, platelets, sCr, or BUN were missing within the ± 96 hour period.

**Test Cohort**

Validation of the identified screening tool was completed in 370 retrospective matched cases (n=740) > 65 years of age (120 matched cases (n=240) were > 80 years old and 250 matched cases (n=500) were between 65 – 79 years of age). Criteria for data collection were the same as those used in the development of the screening tool for all parameters that remained in the final screening tool, but with an allowance of up to 7 days before study entry date permitted for laboratory parameters in the identified BSI screening tool (BUN, PMN, Glucose, albumin, and ALT).

**Table S1. Learn Cohort Types and Sources of Bacteremia**

| **Bacteria** | **Number of Isolates** | **% of Isolates** |
| --- | --- | --- |
| *E. coli*^a^ | 37 | 34 |
| *S. aureus*^b^ | 22 | 20 |
| *Enterococcus spp.* | 7 | 6 |
| B-hemolytic *Streptococci* | 7 | 6 |
| *K. pneumoniae* | 6 | 5 |
| *S. pneumoniae* | 5 | 5 |
| *E. cloacae* | 4 | 4 |
| *P. aeruginosa* | 3 | 3 |
| *B. fragilis* | 3 | 3 |
| Other^c^ | 16 | 15 |
| Total Isolates | 110 | 101^d^ |
| **Monomicrobial vs. Polymicrobial Bacteremia** | **Number of Patients** | **% of Patients** |
| Monomicrobial | 100 | 95 |
| Polymicrobial (2 non-contaminant bacteria)^e^ | 5 | 5 |
| **Concomitant Source with Positive Culture** | **Number of Patients^f^** | **% of Patients** |
| Urine^g^ | 48 | 46 |
| Joint Fluid | 5 | 5 |
| Respiratory | 3 | 3 |
| Soft Tissue | 2 | 2 |
| CVC | 1 | 1 |
| Hepatic/biliary | 2 | 2 |

^a^Three patients has Extended Spectrum Betalactamase producing E. coli

^b^One patient had 2 strains: Methicillin Sensitive *S. aureus* and Borderline Resistant *S. aureus*

^c^Includes all isolates with a prevalence of ≤ 2% (2% each: *S. gallolyticus, Morganella morganii, Clostridium perfringens, Citrobacter koseri, S. anginosus*; 1% each: *Raoultella planticola, Listeria monocytogenes, Fusobacterium nucleatum, S. mitis, Peptostreptococcus micros, K. oxytoca*)

^d^Total Percent sums to >100% due to rounding

^e^No patient had a blood culture with >2 non-contaminant bacteria

^f^Patients may have had >1 concomitant positive source

^g^65% (31/48) were the same as blood culture

**Table S2. Comparison of Bacteremia Screening Tools Developed in Learn Cohort^a^**

| **Tool** | **Sensitivity (%)** | **Specificity (%)** | **Accuracy (%)** | **Pre-test Probability (%)** | **Positive Predictive Value (also positive post test probability) (%)** | **Negative Predictive Value (%)** | **Negative post test probability** | **Positive Likelihood Ratio** | **Negative Likelihood Ratio** | **False Positive Rate (1-specificity) (%)** | **False Negative Rate (1-sensitivity) (%)** |
| --- | --- | --- | --- | --- | --- | --- | --- | --- | --- | --- | --- |
| **Optimal Binary Logistic Regression Equation with Probability Threshold identifying Infection of >0.5 (N=130, Cases=73, Controls=57)** | **94.5** | **94.7** | **94.6** | **56.2** | **95.8** | **93.1** | **6.9** | **18.0** | **0.06** | **5.3** | **5.5** |
| **CART Simplest Tree from Input of BLR signficant parameters (If Temperature >37.55 OR Temperature </= 37.55C and PMN >11.65 then BSI) (N=210, Cases=105, Controls=105)** | **87.6** | **93.3** | **90.5** | **50.0** | **92.9** | **88.3** | **11.7** | **13.1** | **0.13** | **6.7** | **12.4** |
| **CART selected split when all BLR significant parameters input and parent node is Temperature (Temperature >37.55 AND PMN>7.95)**  **(N=210, Cases=105, Controls=105)** | **52.3** | **99.0** | **75.7** | **50.0** | **98.2** | **67.5** | **32.5** | **55** | **0.48** | **0.95** | **47.6** |
| **Tool using CART identified breakpoints for all 7 Significant Parameters identified in optimal BLR tool and each parameter given equal weighting (Tmax>37.55, PMN>7.95, change in LOC=1, BUN>10.05, Glucose>7.35, Albumin ≤33.5, ALT>19.5) (N=130, Cases=73, Controls=57) Number of Parameters that achieved CART breakpoint:** |  |  |  |  |  |  |  |  |  |  |  |
| **Zero (N=14, Cases=0, Controls=14)** | **0.0** | **75.4** | **33.1** | **56.2** | **0.0** | **37.1** | **62.9** | **0.0** | **1.33** | **24.6** | **100.0** |
| **1 (N=23, Cases=2, Controls=21)** | **2.7** | **63.2** | **29.2** | **56.2** | **8.7** | **33.6** | **66.4** | **0.07** | **1.54** | **36.8** | **97.3** |
| **2 (N=15, Cases=2, Controls=13)** | **2.7** | **77.2** | **35.4** | **56.2** | **13.3** | **38.3** | **61.7** | **0.12** | **1.26** | **22.8** | **97.3** |
| **3 (N=22, Cases=15, Controls=7)** | **20.6** | **87.7** | **50.0** | **56.2** | **68.2** | **46.3** | **53.7** | **1.67** | **0.91** | **12.3** | **79.5** |
| **4 (N=25, Cases=23, Controls=2)** | **31.5** | **96.5** | **60.0** | **56.2** | **92.0** | **52.4** | **47.6** | **9.0** | **0.71** | **3.5** | **68.5** |
| **5 (N=18, Cases=18, Controls=0)** | **24.7** | **100.0** | **57.7** | **56.2** | **100.0** | **50.9** | **49.1** | **∞** | **0.75** | **0.0** | **75.3** |
| **6 (N=12, Cases=12, Controls=0)** | **16.4** | **100.0** | **53.1** | **56.2** | **100.0** | **48.3** | **51.7** | **∞** | **0.84** | **0.00** | **83.6** |
| **7 (N=1, Cases=1, Controls=0)** | **1.4** | **100.0** | **44.6** | **56.2** | **100.0** | **44.2** | **55.8** | **∞** | **0.99** | **0.00** | **98.6** |

**^a^Number of patients included in each tool were those with a complete data set for the tool and are indicated in brackets with each tool row.**

**Table S3. Patient Demographics, Microbiology Summary and Tool Performance of Patients with Full Data Set Included in the Bloodstream Infection Tool Analyses**

**Table S4. What would happen to Positive Predictive Value, Negative Predictive Value and Negative Post-Test Probability if the true Pre-test Probability for Bacteremia in Older Patients was Higher or Lower than the Study Pre-Test Probability?^a^**

| If Pre-test Probability (%): | Positive Predictive Value (%) | Negative Predictive Value (%) | Negative Post-Test Probability (%) | |
| --- | --- | --- | --- | --- |
| 1 | 7 | 99.7 | 0.3 | |
| 2^b^ | 12 | 99.5 | 0.5 | |
| 3 | 18 | 99.2 | 0.8 | |
| 4 | 23 | 99 | 1 | |
| 5 | 27 | 99 | 1 | |
| 10 | 44 | 97 | 3 | |
| 15 | 55 | 96 | 4 | |
| 20 | 64 | 94 | 6 | |
| 25 | 70 | 92 | 8 | |
| 30 | 75 | 90 | 10 | |
| 35 | 79 | 88 | 12 | |
| 40 | 82 | 85 | 15 | |
| 45 | 85 | 82 | 18 | |
| 50 | 87 | 79 | 21 | |
| 55 | 90 | 76 | 24 | |
| 60^c^ | 91 | 72 | 28 | |
| 70 | 94 | 62 | 38 | |
| 80 | 97 | 49 | 51 | |
| 90 | 98 | 30 | 70 | |
| 100 | 100 | 0 | 100 | |
|  |  |  |  |  |
| ^a^Sensitivity and Specificity Metric Data for Validation Cohort of Patients ≥ 65 years old was used for calculations in this table | | | | |
| ^b^ Pre-test probability of bloodstream infection for patients ≥ 65 years old at study hospital during study period | | | | |
| ^c^ Study population pre-test probability for validation cohort patients ≥ 65 years old for verification of table functionality | | | | |
|  |  |  |  |  |
